# Iron Deficiency and Oral Treatments: Limitations, Pharmacokinetics, and the Role of Iron Protein Succinylate in Clinical Practice

**DOI:** 10.3390/jcm15103691

**Published:** 2026-05-11

**Authors:** José Antonio García-Erce, Santiago García-López, Antonio Martínez-Francés

**Affiliations:** 1 Blood and Tissue Bank of Navarra, 31008 Navarra, Spain; 2 Gastroenterology Department, Hospital Universitario Miguel Servet, 50009 Zaragoza, Spain; 3 Department of Hematology, Hospital Clínico Universitario Santa Lucía, 30202 Cartagena, Spain

**Keywords:** ID, oral iron supplementation, iron protein succinylate, hepcidin, iron absorption, tolerability

## Abstract

Iron deficiency (ID) is the most prevalent nutritional disorder worldwide, affecting diverse populations including children, women of reproductive age, older adults and patients with chronic conditions. Oral iron supplementation remains the cornerstone of treatment, together with management of the underlying causes. However, conventional ferrous and ferric salts are often associated with gastrointestinal side effects, poor adherence and limited efficacy, especially in inflammatory settings due to hepcidin-mediated absorption blockade. This review summarizes iron absorption physiology, limitations of traditional oral therapies, and the potential benefits of iron protein succinylate (IPS), a ferric complex bound to succinylated casein. IPS provides pH-dependent release and contains succinic acid, which may enhance absorption while reducing gastrointestinal adverse events. Clinical studies indicate that IPS achieves hematologic outcomes comparable or superior to standard oral salts, fewer gastrointestinal effects and better tolerability. These properties make IPS suitable for patients who do not tolerate or respond adequately to conventional therapy. Special attention is given to chronic inflammation, pregnancy, cancer, or gastrointestinal disorders, where oral iron often fails due to impaired absorption or poorer tolerance. Practical recommendations are included to optimize supplementation through dosing strategies and tailored approaches. Overall, IPS offers an effective, better-tolerated alternative to conventional oral iron therapy.

## 1. Introduction

Iron deficiency (ID), defined by the World Health Organization (WHO) as a condition in which there are no mobilizable iron stores and signs of a compromised supply of iron to tissues, including the erythron, are noted [1], is the most common nutritional deficiency worldwide and a major contributor to global morbidity. In 2021, it affected over 1.27 billion individuals, and projections suggest 1.44 billion cases by 2050. Prevalence varies by age, sex, physiological status, geography, and socioeconomic conditions, with women of reproductive age and children under five years among the most affected groups [2]. Causes and contributing factors include inadequate intake, malabsorption, chronic blood loss, and increased demands related to growth, menstruation, or pregnancy [3,4]. Additional contributors include chronic inflammatory diseases, gastrointestinal disorders or bleeding, and medications that impair absorption [5,6,7].

From the earliest stages of iron depletion, symptoms can emerge due to its involvement in energy metabolism. In adults, early deficiency is characterized by fatigue and higher healthcare utilization [8]. Women of reproductive age are especially affected, with up to 40% showing depleted stores due to menstrual losses and suboptimal intake [5]. During pregnancy, iron requirements triple to support fetal growth, placental function, and maternal red cell expansion [3], increasing the risk of maternal morbidity, and peri- and postpartum consequences [3,4,9]. Inadequate maternal iron also threatens fetal neurodevelopment and is linked to cognitive, motor, and socioemotional impairments in the child [10]. Infants and young children are also frequently affected due to rapid growth, limited stores, and insufficient intake [11]. In Europe, up to 25% of children under age three suffer from ID [11], leading to impairments in attention, motor and learning development, and reduced immunity, increasing morbidity and mortality [4,10,12].

Another major consequence of ID is ID anemia, regarded as a subset and the most advanced stage of iron deficiency, in which iron-deficient erythropoiesis reduces hemoglobin concentrations below optimal levels [3,4,5,13]. Anemia markedly increases risk, as seen in pregnancy, where severe forms double the likelihood of maternal death around delivery [9]. The WHO highlights that blood should be considered an organ essential for oxygen transport, immune defense, and hemostasis, and reports that anemia affects nearly 2 billion people worldwide, while ID impacts over 1 billion [14,15]. Despite this burden, both conditions remain underrecognized and poorly managed, with substantial global health consequences. Frequency does not imply normality, and for this reason, the WHO states that anemia in older adults should be considered as a pathological and treatable condition [14,16]. ID and anemia are also underrecognized in patients with chronic inflammation, cancer, or recent surgery [6,8,17,18], supporting early identification and management tailored to severity and comorbidities [8].

For these reasons, iron supplementation is the cornerstone of ID management, together with identifying and treating its underlying cause. In addition, formulation choice depends on the severity of the condition, patient characteristics, and tolerability. Intravenous (IV) iron therapy is indicated in patients with poor gastrointestinal tolerance, absorption disorders, or moderate to severe anemia [8]. IV iron is particularly beneficial in chronic inflammatory diseases, cancer, or when rapid recovery from anemia is needed, including patients with vascular pathology or scheduled for early surgery [6,7,8,17,19]. Nonetheless, oral iron remains first-line in most cases due to its availability and affordability [3]. Common oral options include ferrous iron salts, polysaccharide complexes, amino acid complexes or chelates, iron protein succinylate (IPS), nanoparticle or lipophilic iron chelators, and heme-iron polypeptides, mainly absorbed in the duodenum [3]. Table 1 summarizes the active iron compounds currently available in Europe, including oral and parenteral formulations authorized for clinical use. Despite their widespread use, some oral iron formulations are limited by gastrointestinal side effects, which can compromise adherence. In addition, their absorption may be reduced in chronic inflammatory conditions, where elevated hepcidin levels block ferroportin-mediated iron export from enterocytes [8,18]. Among oral options, IPS has shown efficacy comparable to or greater than other formulations, while reducing adverse events [4,20]. Its stability in the digestive tract may enhance absorption and tolerability, supporting its role as one of the most suitable oral alternatives with proven efficacy and safety [4,11].

Overall, ID remains common and clinically significant, highlighting the importance of understanding iron physiology and therapeutic alternatives. In this context, clinical evidence shows that IPS is an effective and well-tolerated option across diverse populations [20]. Building on this rationale, this review will explore iron absorption and regulation, limitations of conventional oral therapy, and the pharmacological profile of IPS, emphasizing its efficacy and tolerability in clinical practice.

## 2. Iron Physiology and Metabolism

Iron is an indispensable trace element, with an average adult body containing approximately 3.5 g, of which roughly 2.1 g are sequestered in hemoglobin, while the remainder supports intracellular proteins (such as myoglobin and cytochromes) and circulates bound to transferrin. Because humans lack a mechanism for active iron excretion, systemic homeostasis depends on tightly regulated intestinal absorption and the reutilization of endogenous stores [21,22,23].

Dietary iron absorption takes place mainly through enterocyte cells located in the duodenum and the upper jejunum of the small intestine, where absorption capacity is inherently limited [24]. In humans, the average daily dietary iron intake is approximately 10–15 mg, of which only 1–2 mg are typically absorbed through the intestinal mucosa, even under conditions of ID [24]. Iron is available in two chemically distinct forms, non-heme and heme, each following specific uptake and transport pathways. Non-heme iron, predominantly derived from plant-based foods, is present as ferric iron (Fe^3+^) and must be reduced to the more soluble ferrous form (Fe^2+^) by duodenal cytochrome b before uptake via the divalent metal transporter 1 (DMT1) [24,25]. Once inside enterocytes, Fe^2+^ may be sequestered in ferritin or exported across the basolateral membrane by ferroportin; immediately upon export, iron binds circulating transferrin to ensure systemic delivery. In contrast, heme iron, abundant in animal-based foods, is absorbed intact via the heme carrier protein 1 (HCP1); intracellularly, heme oxygenase cleaves the porphyrin ring to release Fe^2+^, which then follows the same ferritin-storage or ferroportin-export pathway as non-heme iron [25]. Accessory proteins further optimize these processes: mucins maintain ferric iron solubility under acidic luminal conditions, brush-border integrins interact with mobilferrin to shuttle absorbed iron within enterocytes, and ferroportin mediates the final basolateral export of both heme-derived and non-heme iron [25,26].

Once in the circulation, iron distribution and storage are centrally regulated by the hepcidin–ferroportin axis. Hepcidin, a 25-amino-acid peptide hormone synthesized predominantly by hepatocytes, binds ferroportin on enterocytes, macrophages (involved in iron recycling), and hepatocytes (responsible for storage), triggering ferroportin internalization and degradation. This interaction decreases iron efflux into the plasma when body iron levels are sufficient [27,28,29]. Since hepatocytes are also the main site of hepcidin production, the liver integrates the principal signals that regulate its expression. In this context, increased circulating and tissue iron stimulates hepcidin synthesis predominantly through the bone morphogenetic protein (BMP)-SMAD pathway, in which BMP2 and BMP6, produced by liver sinusoidal endothelial cells, interact with BMP receptors on hepatocytes together with hemojuvelin, while HFE, transferrin receptor 1 and transferrin receptor 2 contribute to iron sensing and promote HAMP transcription [30]. Inflammatory stimuli also increase hepcidin expression, mainly through the interleukin-6 (IL-6)-Janus kinase (JAK)-signal transducer and activator of transcription 3 (STAT3) pathway, whereas erythropoietic drive and hypoxia suppress hepatic hepcidin production, thereby favoring ferroportin-mediated iron export and intestinal iron absorption [30]. Accordingly, during ID, hepcidin synthesis is suppressed, allowing ferroportin to remain at the cell surface and facilitate the release of stored iron to support erythropoiesis. Conversely, inflammatory cytokines, particularly IL-6, upregulate hepcidin production, inducing hypoferremia by sequestering iron within macrophages and restricting its availability to pathogens [28,31]. Genetic or acquired dysregulation of hepcidin or ferroportin function can lead to clinical disorders ranging from ID anemia to iron overload syndromes such as hereditary hemochromatosis [27,28,29,31].

Assessment of iron status relies primarily on serum ferritin and hemoglobin measurements. Ferritin, considered the gold standard for evaluating iron stores, reflects total body iron reserves; low ferritin levels indicate depleted stores and early ID. Therefore, it serves as an early preventive marker for anemia [32]. Although the optimal ferritin threshold remains debated, a serum ferritin <30 ng/mL is widely used in clinical practice as a sensitive and specific cut-off for absolute ID, improving on the traditional <15 ng/mL threshold proposed in the general WHO criteria [13,33]. However, this threshold should be interpreted according to the clinical context [33]. In patients undergoing evaluation for gastrointestinal disease, the American Gastroenterological Association has proposed a higher cut-off of <45 ng/mL, whereas the WHO recommends a threshold of <70 ng/mL in adults when inflammation or infection is suspected [13,34]. In the presence of confirmed inflammation, ID, with or without anemia, may be diagnosed when ferritin is <100 ng/mL or transferrin saturation (TSAT) is <20%, although a TSAT threshold of <16% has been used in some studies in inflammatory bowel disease [33]. Accordingly, absolute ID is diagnosed by serum ferritin <30 ng/mL and may be accompanied, or not, by anemia [35]. However, as an acute-phase reactant, ferritin may be elevated in inflammatory states, complicating its interpretation in chronic disease or concurrent inflammation [32,36]. In such settings, ID should be assessed using higher ferritin thresholds (commonly <100 ng/mL, and potentially higher in chronic kidney disease and congestive heart failure) together with low transferrin saturation (<20%) [35]. Hemoglobin concentration, which assesses functional iron availability for erythropoiesis, decreases once iron stores are exhausted; although valuable for diagnosing anemia, hemoglobin is not specific to ID, since other conditions (e.g., vitamin deficiencies or blood loss) can also lower its level. The traditional WHO definition of anemia establishes Hb < 13 g/dL in men aged 15–65 years, and Hb < 12 g/dL in nonpregnant women aged 15–65 years [37]. However, an update has been proposed for Caucasian populations based on data from the National Health and Nutrition Examination Survey (NHANES III), suggesting Hb < 13.7 g/dL in men aged 20–59 years, Hb < 13.2 g/dL in men aged ≥60 years, and Hb < 12.2 g/dL in women [16]. In addition, studies in Spanish populations recommend considering altitude more carefully, as the required adjustment may be greater than the WHO correction, approaching 0.5 g/dL above 1000 m [38]. Consequently, combining ferritin and hemoglobin with additional markers, such as soluble transferrin receptor, provides a more comprehensive evaluation of iron status [32].

## 3. Challenges of Conventional Iron Supplementation

**Figure 1:**
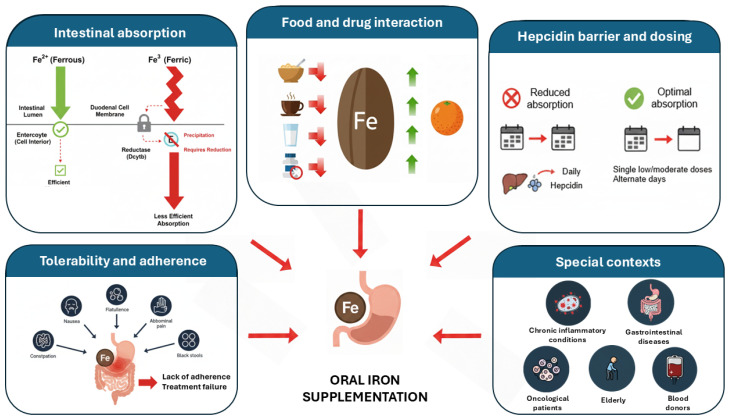
Main barriers associated with conventional oral iron supplementation.

To better contextualize current therapeutic limitations, the following sections describe the main challenges associated with conventional oral iron supplementation, including metabolic, absorption-related, hepcidin-mediated and tolerability barriers, which are summarized in Figure 1.

### 3.1. Metabolic Barriers to Oral Iron Uptake

Dietary iron and iron contained in oral supplements exist mainly as ferric (Fe^3+^) or ferrous (Fe^2+^) iron, but only the divalent form is efficiently absorbed. Non-heme ferric iron precipitates at the near-neutral pH of the duodenum and must be reduced by brush-border reductases such as duodenal cytochrome b before transport via DMT1, whereas ferrous salts, such as ferrous sulfate, gluconate or fumarate, bypass this redox requirement and achieve fractional absorption of approximately 10–15 % under standard conditions. By contrast, ferric complexes (e.g., iron-polymaltose) exhibit three- to four-fold lower bioavailability due to poor solubility in alkaline media and obligatory reduction to Fe^2+^ [21,24,39].

### 3.2. Absorption Barriers: Food and Drug Interactions

Common dietary components, supplements, and concomitant medications can substantially reduce intestinal iron absorption and, in many cases, diminish the exposure of co-prescribed drugs. Phytic acid in cereals, legumes and nuts chelates non-heme iron in a dose-dependent manner, reducing absorption by 18–82% [24]. Polyphenols from tea, coffee, wine, fruits and vegetables form insoluble complexes with ferric iron, lowering uptake by up to 85% [39]. Calcium and antacids, by raising gastric pH or competing at the enterocyte, can halve single-meal iron absorption [24]. Co-administration with ferrous sulfate markedly reduces antibiotic and other drug levels: tetracyclines by 50–90%, penicillamine by 80%, methyldopa by 61–73%, levodopa by 51% (and its carbidopa component by >75%), and ciprofloxacin’s peak and total exposure by 75% and 64%, respectively. Therefore, to maximize iron uptake and preserve co-prescribed drug efficacy, oral iron should be administered at least 2–3 h apart from inhibitory foods, supplements and medications [40]. To enhance efficacy and improve adherence, recent guidelines suggest taking iron at bedtime with water, a strategy that helps avoid dietary inhibitors such as dairy products, phytates, caffeinated beverages, and calcium-rich juices. When appropriate, co-administration with vitamin C is also recommended, as 500 mg of ascorbic acid may facilitate iron absorption even in the presence of calcium or fiber in the meal [34].

### 3.3. Hepcidin-Informed Dosing Constraints

Emerging evidence shows that hepcidin dynamics should guide dosing, favoring single low to moderate doses on alternate days over conventional daily or twice-daily regimens to maximize net absorption and tolerability. Conventional oral regimens, typically 60–120 mg elemental iron daily, trigger a counter-regulatory increase in the iron-regulatory hormone hepcidin, which degrades ferroportin and sharply inhibits subsequent absorption. In iron-depleted women, single doses ≥ 60 mg raise serum hepcidin for 24 h and reduce next-day absorption by 35–45% (*p* < 0.01), while twice-daily dosing offers no net gain despite higher intake [41]. Over 14 versus 28 days of 60 mg ferrous sulfate administration on consecutive versus alternate days, cumulative absorption was 16.3 % versus 21.8 % (*p* = 0.0013) and total uptake 131 mg versus 175 mg (*p* = 0.0010), with significantly higher hepcidin in the daily group (*p* = 0.0031), underscoring the advantage of single, low-to-moderate doses on alternate days [42]. This chronic elevation of hepcidin renders oral iron particularly ineffective in these patients, as iron is rapidly sequestered and fails to reach the sites where it is needed, a phenomenon that has been described in previous publications [43,44].

### 3.4. Gastrointestinal Tolerability and Adherence Barriers

Dose-dependent gastrointestinal symptoms are the leading cause of poor adherence and treatment failure in oral iron therapy. Nausea, flatulence, abdominal pain, diarrhea, constipation and black stools occur in up to 60 % of recipients and represent the principal cause of non-adherence, with up to half of patients discontinuing or skipping doses in response to intolerance [21,45,46].

### 3.5. Special Clinical Contexts in ID: Inflammation, Malabsorption, Cancer, Older Adults, and Blood Donation

**Figure 2:**
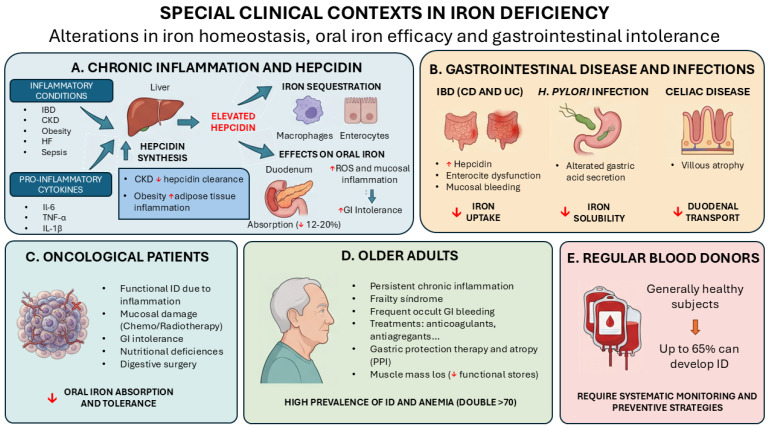
Special clinical contexts in iron deficiency associated with alterations in iron homeostasis, reduced oral iron efficacy, and gastrointestinal intolerance. Abbreviations: CD, Crohn’s disease; CKD, chronic kidney disease; GI, gastrointestinal; HF, heart failure; IBD, inflammatory bowel disease; ID, iron deficiency; IL-1β, interleukin-1 beta; IL-6, interleukin-6; PPI, proton pump inhibitor; ROS, reactive oxygen species; TNF-α, tumor necrosis factor alpha; UC, ulcerative colitis.

Several settings warrant particular attention because they alter iron homeostasis, reduce the effectiveness of oral iron, or heighten gastrointestinal intolerance (Figure 2). In chronic inflammatory conditions such as inflammatory bowel disease, chronic kidney disease (CKD) and obesity, hepatic hepcidin overexpression promotes iron sequestration in macrophages and enterocytes. As a result, oral iron absorption may be reduced to 10–20%, and gastrointestinal intolerance may worsen [47]. Moreover, pro-inflammatory cytokines, notably interleukin-6 (IL-6), tumor necrosis factor-alpha (TNF-α), and interleukin-1 beta (IL-1β), which are elevated in these inflammatory states, play a crucial role in promoting hepatic production of hepcidin [43,44,48,49,50].

This problematic mechanism is amplified in a condition-dependent manner. In CKD, persistent inflammation and reduced hepcidin clearance lead to functional ID and make oral iron ineffective, favoring IV administration [44,48]. In heart failure, chronic inflammation limits iron availability to active tissues, again reducing the efficacy of oral iron [43]. In obesity, adipose-tissue inflammation and IL-6 overproduction elevate hepcidin levels, contributing to hypoferremia, reduced iron absorption, and poor response to oral iron, especially in children [49]. This impaired absorption often necessitates weight loss or probiotic co-therapy, while unabsorbed luminal iron generates reactive oxygen species and mucosal injury, exacerbating gastrointestinal intolerance and leading to treatment discontinuation [51]. In sepsis, systemic inflammation disrupts iron metabolism, but hepcidin and standard biomarkers are unreliable in detecting ID anemia [52].

In this context, inflammatory gastrointestinal diseases such as Crohn’s disease and ulcerative colitis are prototypical examples of iron malabsorption driven by chronic inflammation [18]. These conditions involve increased hepcidin levels, enterocyte dysfunction, and frequent mucosal bleeding, all contributing to reduced iron uptake and anemia of chronic disease [18]. Iron absorption is also compromised in non-inflammatory disorders. For example, *Helicobacter pylori* infection can reduce iron solubility by altering gastric acid secretion, while celiac disease leads to villous atrophy and impaired duodenal transport [6].

Similarly, in oncological patients, inflammation-induced functional ID is common, particularly during chemotherapy or in the presence of tumor-related systemic inflammation [53]. However, additional factors such as mucosal damage from chemotherapy or radiotherapy, gastrointestinal intolerance, nutritional deficiencies, and a history of digestive surgeries may further impair oral iron absorption and tolerability [54].

Another group frequently affected by chronic inflammation is the elderly. This persistent inflammatory state is closely linked to impaired iron homeostasis and the development of frailty syndrome [55]. In addition, the frequent occurrence of occult gastrointestinal bleeding and chronic anticoagulant or/and antiaggregant treatment, along with gastric protection therapy and gastric atrophy (due to chronic proton pump inhibitor use), in this population further contributes to ID and must be assessed based on the patient’s overall clinical condition and diagnostic tolerability [56]. Adding to these factors, age-related loss of muscle mass reduces functional iron stores, exacerbating the risk of ID [57]. Thus, considering the variety of contributing factors, the high prevalence of ID, the higher prevalence of anemia increasing with age and doubling after the seventh decade, and the increasing life expectancy, special attention should be given to this population [16,38].

Lastly, though equally important, an additional challenge lies in not overlooking regular blood donors. Despite their generally healthy status, up to 65% may develop ID, underscoring the need for systematic monitoring and the implementation of preventive treatment strategies in this population [20].

## 4. IPS: Oral Solution with a Specific Mechanism of Action

IPS is characterized by its unique composition: it contains trivalent iron (Fe^3+^) bound to succinylated casein [58,59,60]. The succinylation process involves adding carboxylic groups, which significantly enhances the protein’s negative charge and improves its solubility and iron-binding capacity [61]. Previously published studies indicate that the iron in IPS is present in a trivalent state within a complex structure, assembled into small polymeric clusters, some even below 10 nm, playing a crucial protective role [60]. Also, IPS contains succinic acid, an organic acid that improves iron absorption up to 20–30% [58].

The mechanism of action for IPS is specifically designed to minimize gastrointestinal side effects and optimize absorption through a controlled release pathway. This is made possible, in part, by the fact that the whole complex precipitates in acidic environments [59], specifically at pH levels < 4, preventing the direct release of large concentrations of potentially aggressive iron ions and avoiding direct contact between the iron and the gastric mucosa [58]. This protective mechanism is believed to be a key factor in reducing the gastrointestinal adverse effects commonly associated with other oral iron supplements [58,59,60,61,62]. Furthermore, clinical and pharmacological evidence indicates that IPS maintains its efficacy and absorption even when co-administered with H_2_-receptor antagonists or antacids, since its pH-dependent release prevents iron liberation in the stomach and ensures intestinal absorption remains unaffected [20,63,64].

Moreover, after passing through the stomach, the IPS complex reaches the duodenum and proximal jejunum, where the pH shifts to a neutral or alkaline environment (pH ≃ 7), where the complex becomes soluble again [59,60,65]. Subsequently, the protein matrix is hydrolyzed by pancreatic enzymes, such as trypsin and pancreatin, releasing iron into the gut lumen [58,66,67,68]. There, trivalent iron (Fe^3+^) is reduced to ferrous iron (Fe^2+^) by a membrane ferri-reductase and then transported into enterocytes via the divalent cation transporter DCT1 [60]. In addition, studies indicate that IPS has an open structure that allows for rapid release of iron to complexing agents, facilitating its bioavailability [60]. In vivo evidence supports this efficient absorption process, with an average calculated iron uptake of approximately 18.7% observed in blood donors treated with IPS [62].

One of the main components influencing the absorption of iron from IPS is succinic acid, which plays a key role in enhancing iron uptake. Evidence indicates that the absorption-promoting effect of succinic acid depends on its concentration. Studies conducted with ferrous succinate have shown a dose-dependent improvement in iron absorption [69], while experiments using elemental iron in the form of ferrous sulfate demonstrated a significant increase in absorption when ≥60 mg of succinic acid were added to iron solutions [70]. Importantly, this effect is not simply due to its acidic nature. Other structurally related organic acids, such as α-ketoglutaric, malic, or citric acid, do not produce similar enhancements in absorption [70]. In fact, citric acid may even reduce iron uptake due to complex formation [70]. Moreover, succinic acid’s action does not appear to involve redox activity, as its effect remains unchanged when administered alongside ascorbic acid, a known reducing agent [70]. It also has no observable impact on intestinal motility, ruling out enhanced transit or increased mucosal surface area as potential mechanisms [70]. Given that succinic acid enhances iron absorption even when administered intravenously, when its presence in the intestinal lumen is minimal, it has been hypothesized that it may act through intracellular metabolic pathways, possibly by modulating mucosal iron transport at the cellular level [69,70].

## 5. Comparative Benefits of IPS in Clinical Studies

### 5.1. Optimized Hematologic Efficacy and Bioavailability with Shorter Treatment

Despite containing ferric iron, which is often considered less bioavailable than ferrous forms, IPS demonstrates comparable or even superior absorption efficiency. This may be explained primarily by its pH-sensitive release mechanism [58,59,60,61,62] and the enhancing effect of succinic acid on iron uptake [58,59,69,70].

Clinical evidence accumulated over the past decades demonstrates that IPS achieves effective hematologic correction in patients with ID and anemia across diverse populations. To date, IPS has been evaluated in 54 clinical studies involving approximately 6450 patients, providing robust evidence of its efficacy and safety across a wide range of clinical settings [58]. Early randomized controlled clinical trials established its efficacy and absorption profile relative to conventional ferrous salts (Table 2). In the pharmacokinetic study by Di Giacomo et al. (1987), IPS showed similar iron absorption (6.1%) to ferrous sulfate (5.9%) but produced a steadier increase in serum iron levels over six hours [71]. Likewise, in a controlled trial of blood donors with depleted iron stores, Landucci et al. (1987) reported a threefold higher iron absorption with IPS (18.7%) compared to ferrous sulfate (6.4%), accompanied by a significant rise in serum iron concentration (*p* < 0.001) [62].

In later clinical trials, IPS demonstrated comparable or superior efficacy to slow-release ferrous sulfate. The pilot study by Bregani et al. (1990) found equivalent hematologic recovery between IPS and slow-release ferrous sulfate, with a mean daily hemoglobin gain of 0.055 g/dL for IPS versus 0.048 g/dL for the ferrous formulation [72]. In a larger, single-blind comparison, Pogliani et al. (1990) reported that IPS at doses of 80 mg and 120 mg per day induced greater hemoglobin increases (0.055 and 0.070 g/dL/day, respectively) than slow-release ferrous sulfate (0.048 g/dL/day), and that the higher IPS dose resulted in significantly greater plasma iron concentrations at day 60 (*p* < 0.05) [73]. Similarly, Najean et al. (1995) confirmed the therapeutic equivalence of IPS and ferrous sulfate in a double-blind, multicenter phase III trial including 174 patients, where both treatments corrected anemia and restored iron stores within 60 days [74].

In addition, different trials supported the long-term efficacy of IPS. In a six-month comparative study, Pujol Farriols et al. (2002) found no significant differences in hemoglobin (*p* = 0.39) or ferritin (*p* = 0.37) between IPS and controlled-release ferrous sulfate, with normalization achieved in 83% and 87% of patients, respectively [75]. In women of reproductive age, Marcacci et al. (1989) demonstrated that IPS induced significantly greater increases in hemoglobin and red blood cell indices than ferrous sulfate (*p* < 0.01), together with higher iron absorption (17.7% vs. 10.7%) [76].

The efficacy of IPS has been well demonstrated in patients with gastrointestinal disorders, a population in which oral iron therapy is often limited by malabsorption and intolerance. In the multicenter study by Liguori et al. (1993), after 60 days of treatment, both IPS (120 mg Fe^3+^) and controlled-release ferrous sulfate (105 mg Fe^2+^) normalized hematologic parameters; however, IPS achieved higher final values of hemoglobin, hematocrit, and ferritin, with clinical response rates of 78.9% versus 67.6% [77]. Other oral formulations, such as sucrosomial iron, have also demonstrated efficacy in improving hemoglobin levels in patients with gastrointestinal disorders, such as inflammatory bowel disease. In a 12-week study involving 29 patients with IBD treated with sucrosomial iron 30 mg/day, mean hemoglobin increased significantly from 11.67 ± 0.5 g/dL at baseline to 12.38 ± 0.8 g/dL at the end of treatment (*p* < 0.0001), with anemia correction achieved in 59% of participants. However, mean serum ferritin showed no significant change (45.79 ± 78.8 ng/mL to 44.62 ± 70.8 ng/mL; *p* = 0.798), suggesting that, while sucrosomial iron can correct anemia, it may not fully restore iron stores [78]. Since these formulations have emerged in recent years, additional studies are still needed to confirm their clinical benefits; however, current evidence suggests that these newer ferric formulations may be less effective in fully replenishing iron stores in certain clinical settings.

Furthermore, sucrosomial iron is not recognized by the European Medicines Agency or national medicines agencies as a medication, but it is considered a nutritional supplement. Consequently, the evidence for its benefit and safety in treating ID has not undergone the rigorous evaluation of these agencies [79]. Overall, the primary disadvantages of sucrosomial iron are its high cost and its inability to efficiently rebuild or replenish iron stores [47].

Evidence in obstetric populations was more heterogeneous. In early pregnancy, Minganti et al. (1995) observed comparable changes in transferrin, red blood cell indices, and mean corpuscular parameters between treatments, with ferri-mannitol-albumin showing a significantly greater post-treatment increase in hemoglobin (*p* = 0.0146) compared to IPS [80]. In contrast, during later gestation, Rayado et al. (1996) demonstrated that IPS effectively prevented gestational anemia, maintaining 96.5% of pregnant women non-anemic at term, with hemoglobin and erythrocyte values comparable to those obtained with ferrimanitol–ovoalbumin [63].

In addition, a double-blind, controlled trial (N = 1095) showed both IPS and ferrous sulphate were effective in the treatment of ID. While both normalized hematologic parameters, IPS yielded significantly greater final values for hemoglobin and ferritin, indicating a steadier effect. IPS’s tolerability was significantly superior (11.5% adverse events vs. 26.3% for ferrous sulphate) [77]. These results are supported by a study in which IPS (80 mg Fe^3+^) significantly increased serum iron levels (from 58.5 ± 24.0 µg/100 mL to 81.15 ± 24.6 µg/100 mL, *p* < 0.001) and ferritin levels (from 22.15 ± 14.4 ng/mL to 47.3 ± 27.4 ng/mL, *p* < 0.001) after one month in blood donors with ID, whereas ferrous sulfate (105 mg Fe^2+^) showed no significant changes in serum iron [62].

In terms of treatment duration, preclinical findings by Urso et al. demonstrated that IPS was as effective as ferrous sulphate in fully restoring physiological levels of hemoglobin, hematocrit, and erythrocyte count within just 14 days [59]. The study also reported that IPS did not induce serum hepcidin, whereas FeSO_4_ nearly doubled its levels, although this potential clinical advantage has not been confirmed in humans and requires further studies to be corroborated.

Additionally, the clinical efficacy of IPS may be partially explained by its bioavailability profile. IPS has also demonstrated favorable bioavailability and good absorption, with studies showing its absorption to be comparable to that of iron gluconate and only slightly lower (7–9%) than that of FeSO_4_, which is generally considered more bioavailable than ferric salts [81]. This is supported by clinical data reporting, in general, an increase in serum iron levels from the first 30 min after IPS administration [79,81,82], maintained for an average of 4 to 6 h [82,83,84]. For instance, in blood donors, daily intake of 80 mg Fe^3+^ for one month raised serum iron from 58.5 ± 24.0 to 81.15 ± 24.6 µg/100 mL (*p* < 0.001) [62], and in another study, a single dose increased serum iron from 54.8 ± 30.1 to 103.7 ± 55.6 µg/100 mL within 3 h (*p* < 0.01) [79]. The succinic acid component of IPS may contribute to this effect by enhancing iron absorption; some studies have reported increases in absorption (from 1.03 ± 0.04 to 1.57 ± 0.19, ratio of absorption) when succinic acid was co-administered with ferrous sulfate [70]. However, other findings have been inconsistent, with no significant differences observed in certain studies [69,85], suggesting that the impact of succinic acid on iron absorption may vary depending on dose and context.

Notably, IPS offers a significant advantage over other oral iron formulations, as its absorption is largely unaffected by food intake, which may improve treatment adherence. A study by Deriu and Mastrantoni (1988) showed no statistically significant differences (*p* > 0.05) in serum iron levels when IPS was administered either on an empty stomach or after a meal, with peak values of 135 ± 15 µg/100 mL and 138 ± 17 µg/100 mL at 2 h, respectively [86]. In contrast, the absorption of other ferrous or ferric salts can decrease by approximately 40% when taken with food [87], making IPS a more flexible option for patients. Hence, IPS offers a significant advantage over other oral iron formulations, since its absorption is largely unaffected by food intake, which may improve treatment adherence.

**Table 2:** Summary of clinical studies comparing oral IPS with different iron formulations.

Study	Design	Treatment Arms	Population	Variables	Efficacy Outcomes	Safety Outcomes
**Di Giacomo et al., 1987** [71]	Randomized open-label clinical trial	A: IPS 80 mg Fe^3+^ daily; B: Ferritin complex 80 mg Fe^3+^ daily; C: Ferrous sulfate 105 mg Fe^2+^ daily. Duration: 28 days (efficacy) + 6 h PK sub-study.	40 adults with iron-deficiency anemia (10 per arm for PK, 30 for efficacy).	Serum iron kinetics (7 time points); Hb, SI, TIBC, TSAT; estimated iron absorption.	Ferrous sulfate produced a faster early rise in serum iron, but IPS maintained higher and steadier levels. Slightly higher absorption with IPS (6.1%) vs. FS (5.9%).	N/A
**Landucci et al., 1987** [62]	Randomized open-label controlled clinical trial	A: IPS 80 mg Fe^3+^ daily; B: Ferrous sulfate 105 mg Fe^2+^ daily. Duration: 30 days.	40 blood donors (11 men, 29 women; mean age 36 years) with low ferritin (<30 ng/100 mL).	RBC, Hb, HCT, MCV, SI, transferrin, ferritin; iron absorption (Gordulek formula); tolerability.	IPS significantly increased serum iron (*p* < 0.001) and absorption (18.7%) vs. FS (6.4%). Both improved ferritin, restoring iron stores.	Good overall tolerance; one digestive disorder with IPS and one constipation case with FS.
**Bregani et al., 1990** [72]	Comparative clinical trial	A: IPS 80 mg Fe^3+^ daily; B: Slow-release FS 105 mg Fe^2+^ daily. Duration: 40 days.	40 adults with iron-deficiency anemia (Hb 7–11.5 g/dL, Fe < 50 µg/dL, TSAT < 15%).	Hb increase per day, plasma iron, RBC, HCT; tolerability.	IPS and FS achieved similar Hb recovery (0.055 vs. 0.048 g/dL/day).	IPS significantly better tolerated; fewer GI reactions (*p* < 0.001).
**Pogliani et al., 1990** [73]	Randomized single-blind clinical trial	A: IPS 80 mg Fe^3+^ daily; B: IPS 120 mg Fe^3+^ daily; C: Extended-release FS 105 mg Fe^2+^ daily. Duration: 60 days.	54 adults with iron-deficiency anemia (mean age 57 years): 22, 19, and 13 per arm.	Hb, HCT, SI, RBC; tolerability.	All groups improved; Hb increase greater with IPS. Both IPS doses showed superior tolerability to FS.	Fewer adverse events with IPS; 4 patients discontinued FS due to intolerance.
**Marcacci et al., 1989** [76]	Randomized single-blind clinical trial	Solid forms: IPS (80 mg Fe^2+^/day, chewable) vs. FS (100 mg Fe^2+^/day, capsules). Liquid forms: IPS (80 mg Fe^2+^/day, vials) vs. sodium ferric gluconate (80 mg Fe^3+^/day). Duration: 40 days.	250 women (15–50 years, pregnant/non-pregnant) with iron deficiency or microcytic anemia.	Hb, ferritin, serum iron, HCT, clinical symptoms.	IPS produced greater increases in Hb and RBC vs. FS (*p* < 0.01); iron absorption higher with IPS (17.7% vs. 10.7%).	IPS better tolerated; lowest complaint/benefit ratio (0.52 vs. 2.25).
**Liguori et al., 1993** [77]	Randomized double-blind, double-dummy, multicenter clinical trial	A: IPS 120 mg Fe^3+^ daily (two 60 mg tablets); B: Controlled-release FS 105 mg Fe^2+^ daily. Duration: 60 days.	1095 adults with iron deficiency or iron-deficiency anemia (546 IPS; 549 FS).	Hb, HCT, ferritin; global response; tolerability.	Both normalized Hb and ferritin, but IPS yielded higher increases and higher success rate (78.9% vs. 67.6%).	IPS caused fewer adverse events (11.5% vs. 26.3%), mainly mild GI discomfort.
**Najean et al., 1995** [74]	Randomized double-blind multicenter phase III trial	A: IPS 120 mg Fe^3+^ daily; B: Controlled-release FS 105 mg Fe^2+^ daily. Duration: 60 days.	174 patients (98% women) with mild-to-moderate iron-deficiency anemia.	Hb, HCT, RBC, plasma iron, TSAT, ferritin; clinical symptoms; safety.	Both treatments equally effective for anemia correction.	IPS better tolerated; fewer GI symptoms (*p* < 0.05), especially diarrhea (10% vs. 25%).
**Pujol Farriols et al., 2002** [75]	Comparative observational clinical study	A: IPS 80 mg Fe^3+^ daily (2 vials/day); B: FS 210 mg Fe^2+^/day first month, then 105 mg/day. Duration: 6 months.	60 adults (30 per group) with chronic iron-deficiency anemia (Hb < 10 g/dL, ferritin < 30 µg/dL).	Hb, ferritin, RBC, MCV; long-term tolerability.	Both achieved Hb normalization (~85%); no significant efficacy difference (*p* > 0.3).	IPS had fewer adverse effects (3% vs. 13%); mild nausea only, no discontinuations.
**Minganti et al., 1995** [80]	Randomized single-blind clinical trial	A: Ferrimannitol-ovoalbumin 80 mg Fe^3+^ daily; B: IPS 80 mg Fe^3+^ daily. Duration: 28 days.	15 pregnant women in first trimester with Hb ≤ 11 g/dL (7 A; 8 B).	Hb, RBC, serum iron, transferrin, MCV, MCH, MCHC; tolerability.	Only ferri-mannitol-albumin showed a significant Hb increase (*p* = 0.0146); final Hb higher than IPS (*p* = 0.023).	Tolerability good or optimal in both; no dropouts for side effects.
**Rayado et al., 1996** [63]	Randomized phase IV comparative clinical trial	A: Ferrimanitol-ovoalbumin 40 mg Fe^3+^ daily; B: IPS 40 mg Fe^3+^ daily. Duration: 13 weeks (weeks 24–32 of gestation).	394 pregnant women (347 evaluable: 172 A; 175 B).	Hb, RBC, HCT, MCV, MCH, ferritin; prevention of gestational anemia; safety.	Both effectively prevented gestational anemia (96.5% non-anemic). Ferritin rose more with ferrimanitol–ovoalbumin (*p* = 0.001); Hb, RBC, HCT, MCV, MCH, similar between groups.	Acceptable tolerability in both groups; very few mild digestive complaints.

**Abbreviations:** AE: adverse event; Fe^2+^: ferrous iron; Fe^3+^: ferric iron; FS: ferrous sulfate; GI: gastrointestinal; Hb: hemoglobin; Hct: hematocrit; IPS: iron protein succinylate; MCV: mean corpuscular volume; MCH: mean corpuscular hemoglobin; MCHC: mean corpuscular hemoglobin concentration; PK: pharmacokinetic; RBC: red blood cells; SI: serum iron; TIBC: total iron-binding capacity; TSAT: transferrin saturation.

### 5.2. Superior Tolerability Compared to Conventional Ferrous and Ferric Salts, in Addition to a Significant Reduction in Gastrointestinal Adverse Effects

IPS also shows an excellent tolerability and safety profile across clinical trials, consistently outperforming conventional ferrous salts in reducing gastrointestinal intolerance and treatment discontinuation. Across randomized controlled clinical trials conducted over three decades (Table 2), no serious adverse events were reported. In the trial by Bregani et al. (1990), IPS showed markedly better tolerability than slow-release ferrous sulfate, with significantly fewer gastrointestinal reactions (*p* < 0.001) [72]. Similarly, Pogliani et al. (1990) reported adverse events in only 4.5% of patients treated with IPS compared to 22% with ferrous sulfate, with treatment discontinuations occurring exclusively in the ferrous group [73]. The large multicenter trial by Liguori et al. (1993) confirmed these findings, showing an adverse event rate of 11.5% for IPS versus 26.3% for controlled-release ferrous sulfate, mainly mild gastrointestinal effects such as heartburn or constipation [77]. Comparable results were described by Najean et al. (1995), who found significantly fewer gastrointestinal symptoms with IPS (*p* < 0.05), including a lower incidence of diarrhea (10% vs. 25%) [74]. In long-term treatment, Pujol Farriols et al. (2002) reported adverse events in 3% of patients receiving IPS versus 13% with ferrous sulfate [75], while Marcacci et al. (1989) observed a complaint-to-benefit ratio almost five times lower for IPS (0.52 vs. 2.25) [76].

In obstetric populations, Minganti et al. (1995) and Rayado et al. (1996) described very favorable safety profiles, with no treatment withdrawals and only isolated mild digestive effects [63,80]. Early evaluations by Di Giacomo et al. (1987) and Landucci et al. (1987) also reported no relevant adverse events [62,71].

In parallel, other clinical studies have reported an excellent safety profile for IPS, with no adverse events observed in participants [81,84]. Additionally, Landucci et al. (1987) reported only one patient per group experiencing mild gastrointestinal symptoms, digestive discomfort in the IPS group and constipation in the FeSO_4_ group, highlighting the good overall tolerability of IPS [62]. This favorable safety and adherence profile has also been observed in patients with gastrointestinal conditions [88], further reinforcing the tolerability advantages of IPS in populations typically more vulnerable to gastrointestinal side effects.

In contrast, conventional oral iron formulations, such as FeSO_4_, are frequently associated with gastrointestinal adverse events including nausea, vomiting, constipation, and diarrhea, reported in 25–40% of patients [45,46]. These effects can lead to treatment failure in up to 50% of cases due to poor adherence [45].

Comparison of IPS and conventional oral iron across efficacy, absorption, and tolerability is summarized in Table 3, and rsubpopulation-specific outcomes are presented in Table 4.

## 6. Optimizing Oral Iron Treatment: Practical Recommendations

Multiple practical recommendations have been proposed regarding the use of oral iron therapy. Below, we include a table with good practices and tips we recommend for ID (Table 5). In relation to treatment, oral iron supplementation is the first-line treatment for ID and ID anemia in most patients [47,89,90]. Many oral iron formulations are available, allowing selection based on the clinical context and individual patient characteristics [47,90]. Current recommendations suggest using lower single daily doses (approximately 40–80 mg of elemental iron) or every-other-day regimens in order to improve both iron absorption and tolerability, since hepcidin can induce an absorption blockade that limits iron uptake and affects tolerability [90]. For children, the usual dosing is 3 to 6 mg/kg/day of elemental iron, often divided into two or three doses [90]. Daily administration is usually sufficient and taking iron more than once per day does not increase efficacy but may worsen tolerability [90]. In cases of poor tolerance, alternate-day dosing may be considered, as recent evidence suggests it may provide comparable efficacy with fewer side effects [34,47].

Although oral iron absorption is facilitated in the fasting state, it often causes gastrointestinal adverse effects that may reduce adherence; therefore, administration must often be adapted, usually in relation to meals to manage adverse events and improve adherence, as some patients will better tolerate taking iron with meals [34,91]. The recommendation regarding the co-administration of Vitamin C (ascorbic acid) with oral iron differs significantly between major guidelines, reflecting a divide between mechanistic theory and clinical outcome data. The American Gastroenterological Association (AGA) explicitly gives the Best Practice Advice to “Add vitamin C to oral iron supplementation to improve absorption”, detailing that Vitamin C works by reducing ferric iron to the more absorbable ferrous form and preventing the formation of insoluble iron compounds; the AGA cites that doses of up to 500 mg can be used [34]. In contrast, the European Hematology Association (EHA) states that the administration of vitamin C is not recommended, basing this strong non-recommendation on a large randomized controlled trial which found that Vitamin C “neither enhances the hematological response nor diminishes the side effects” [92]. In addition, certain substances such as coffee, tea, dairy products, and calcium-rich foods should be avoided within at least one hour of taking iron, as they inhibit absorption [34,90,91].

Hemoglobin levels should be reassessed after 4 weeks of treatment [90]. A rise of at least 1 g/dL in hemoglobin is generally expected within this period if the therapy is effective [47,89,90]. Once the anemia has resolved, oral iron should be continued for at least 3 additional months to ensure repletion of iron stores [90].

Moreover, oral iron use should be tailored according to the characteristics and comorbidities of specific patient populations [47,90]. In pregnant individuals, oral iron is considered the first-line option during the first trimester, provided that it is well tolerated and gastrointestinal absorption is adequate [89]. This route of administration remains appropriate in later stages of pregnancy as long as the patient responds adequately and tolerates the treatment. However, during the second and third trimesters, if moderate to severe anemia is present, oral iron is not tolerated, or hematologic response is insufficient, a switch to IV iron therapy is recommended, as it allows for faster and more effective repletion of iron stores [89].

In elderly patients [16], particularly those with chronic kidney disease or heart failure, oral iron may still be considered as initial therapy [90]. However, diagnostic thresholds for ferritin may require adjustment due to inflammation-related alterations, and gastrointestinal intolerance is common [90]. In patients with chronic inflammation or cancer, oral iron can be trialed in stable cases with mild disease, but its effectiveness may be limited by hepcidin-mediated absorption blockade [34,89,93]. Similarly, after bariatric surgery, oral iron may be less effective due to altered gastrointestinal anatomy and reduced gastric acid production [34]. In all these scenarios, if oral iron is not tolerated or fails to produce an adequate hematologic response, switching to IV iron is recommended [34,89,90]. Treatment decisions should be individualized, taking into account the urgency of repletion, absorption capacity, and patient tolerability, with regular monitoring to guide adjustments [34,89,90].

## 7. Conclusions

ID remains a prevalent and clinically relevant condition affecting 1.5 billion people, with or without anemia, including older adults, pregnant women, children, patients with chronic diseases, and individuals with cancer or gastrointestinal disorders. In these groups, anemia is associated with increased fatigue, reduced functional capacity, and poorer quality of life, often worsening the underlying condition. Addressing ID effectively and safely is therefore a key component of comprehensive care. In this regard, IPS is an oral formulation that has been used in clinical practice for several decades and offers certain advantages over traditional ferrous and ferric salts. Its formulation allows for gradual intestinal release, improved absorption through the action of succinic acid, and good gastrointestinal tolerability. These characteristics are associated with comparable or, in some cases, improved hematologic outcomes, along with a significantly better tolerability, fewer gastrointestinal side effects and flexible administration with food. While no single therapy is suitable for all patients, the clinical experience with IPS supports its use as a therapeutic option in the management of ID. Additionally, these considerations align with the growing emphasis on Patient Blood Management, which promotes the early identification and correction of ID and anemia to optimize patients’ own blood and reduce transfusion requirements. This is particularly important in surgical candidates and pregnancy, in whom anemia is common and linked to higher transfusion rates and longer hospital stays, so preoperative detection and treatment should be prioritized to improve outcomes.

## Figures and Tables

**Table 1:** Iron formulations that are available in Europe.

Active Substance	Chemical Form	Route of Administration
Ferrous sulfate	Fe^2+^	Oral
Ferrous fumarate	Fe^2+^	Oral
Ferrous gluconate	Fe^2+^	Oral
Ferric polymaltose	Fe^3+^	Oral
Iron protein succinylate	Fe^3+^	Oral
Sucrosomial iron	Fe^3+^	Oral
Ferric carboxymaltose	Fe^3+^	IV
Ferric sucrose	Fe^3+^	IV
Ferric isomaltoside	Fe^3+^	IM/IV
Low-molecular-weight iron dextran	Fe^3+^	IM/IV
Ferric gluconate	Fe^3+^	IV

**Abbreviations:** Fe^2+^: ferrous iron; Fe^3+^: ferric iron; IM: intramuscular; IV: intravenous.

**Table 3:** Key differences between IPS and conventional oral iron formulations (ferrous and ferric).

Aspect	IPS vs. Conventional Oral Iron
Iron form and release mechanism [57,58,60,61,70]	IPS (Fe^3+^–succinylated casein) shows pH-dependent release, protecting the gastric mucosa; ferrous and ferric salts cause more epigastric irritation.
Absorption efficiency [57,58,68,69]	IPS demonstrates equal or greater absorption than ferrous sulfate due to controlled release and succinic acid enhancement.
Pharmacokinetics [71]	IPS achieves steady serum iron levels over 6 h, while FeSO_4_ shows sharper but less stable peaks.
In vivo absorption [62]	IPS reached a mean absorption of 18.7%, confirming efficient intestinal uptake.
Short-term hematologic efficacy [72,73]	IPS produced higher Hb gains (0.055–0.070 g/dL/day) than slow-release FeSO_4_ (0.048 g/dL/day).
Multicenter clinical trials [77]	IPS normalized anemia in 60 days, achieving higher Hb, Hct, and ferritin than FeSO_4_ (78.9% vs. 67.6% response).
Therapeutic equivalence [74]	IPS and FeSO_4_ both corrected anemia within 60 days in a double-blind multicenter trial.
Long-term efficacy [75]	IPS and FeSO_4_ achieved similar Hb and ferritin normalization after 6 months (83% vs. 87%).
Reproductive-age women [76]	IPS yielded greater Hb and RBC increases and higher iron absorption (17.7% vs. 10.7%) than FeSO_4_.
Pregnancy outcomes [63,80]	IPS prevented gestational anemia (96.5% non-anemic) with good tolerance; early pregnancy results favored ferri-mannitol-albumin.
Serum iron and ferritin response [61]	IPS 80 mg Fe^3+^ raised serum iron and ferritin significantly; FeSO_4_ showed no relevant change. Sucrosomial iron does not rebuild iron stores.
Treatment duration [57]	IPS reached hematologic correction ~15% faster than ferrous salts (49 vs. 58 days).
Bioavailability dynamics [71,72,73,74,75]	IPS showed absorption similar to iron gluconate and slightly below FeSO_4_ (7–9%).
Effect of food on absorption [86]	IPS absorption unaffected by meals; FeSO_4_ absorption reduced by ≈40% with food.
Tolerability (overall) [72,73,75,77]	IPS showed markedly fewer AEs (3–11%) vs. FeSO_4_ (13–26%), with no serious events.
Pregnancy safety [63,80]	IPS showed excellent safety with no withdrawals; conventional salts caused more GI discomfort.

**Abbreviations:** AE: adverse event; Fe^2+^: ferrous iron; Fe^3+^: ferric iron; FeSO_4_: ferrous sulfate; GI: gastrointestinal; Hb: hemoglobin; Hct: hematocrit; IPS: iron protein succinylate; RBC: red blood cells.

**Table 4:** Subpopulation-specific outcomes with IPS versus conventional oral iron.

Subpopulation	Summary of Outcomes
Women [76]	IPS showed greater improvement in hematologic parameters and better gastrointestinal tolerance than ferrous sulfate.
Pregnant women [63,80]	IPS was comparable in efficacy and safety to conventional iron salts.
Blood donors [62,71]	IPS achieved faster and steadier increases in serum iron and ferritin levels than ferrous sulfate.
Patients with chronic gastrointestinal conditions [75]	IPS demonstrated better gastrointestinal tolerability and lower incidence of adverse effects than ferrous sulfate.

**Table 5:** Summary of good practices and tips for oral iron therapy.

Aspect	Recommendation
Treatment	
Formulation	Selection should consider tolerability and patient context, and it may be useful to ask whether the patient has previously taken oral iron; if they have and tolerated it well, the same formulation can be used.
Dose (adults)	40–80 mg of elemental iron once daily is generally sufficient, while higher doses may be less effective and are certainly less well tolerated.
Dose (children)	3–6 mg/kg/day of elemental iron, divided into 2–3 doses.
Frequency	Once-daily dosing is preferred; avoid more than one dose per day due to increased side effects and hepcidin-mediated reduced absorption.
Alternate-day dosing	May be considered in cases of poor tolerance, with comparable efficacy and better gastrointestinal tolerability.
Food interactions	Iron should preferably be taken between meals or at bedtime; avoid coffee, tea, dairy, calcium-rich foods, and high-phytate meals within at least one hour.
Vitamin C	Co-administration with 80–500 mg of vitamin C may enhance absorption, even in the presence of calcium or fiber.
Time of day	Taking iron at bedtime with water may improve adherence and reduce interference from dietary inhibitors.
Monitoring	
Hemoglobin response	Evaluate hemoglobin after 4 weeks of treatment; an increase of ≥1 g/dL indicates adequate response.
Duration of treatment	Continue for at least 3 months after anemia correction to ensure repletion of iron stores. Treatment discontinuation may be considered once ferritin is >100 ng/mL and transferrin saturation is >20%.
Special populations	
Pregnancy	Oral iron is first-line during the first trimester if tolerated. In the second and third trimesters, switch to IV iron if anemia is moderate/severe or response is inadequate.
Elderly patients	Oral iron may be used initially if tolerated but monitor closely due to frequent gastrointestinal side effects and comorbidities affecting absorption.
Chronic inflammation/cancer	Oral iron can be attempted in mild, stable disease, but is often ineffective in active inflammation due to hepcidin blockade; switch to IV if needed. It is also recommended to review disease-specific guidelines and consensus documents to align with their recommendations regarding iron deficiency management.
Post-bariatric surgery	Oral iron may be less effective due to anatomical and absorptive changes; consider IV iron if response is insufficient.

**Abbreviations:** IV: intravenous; Hb: hemoglobin.

## Data Availability

No new data was generated or analyzed in this study; therefore, data sharing is not applicable.

## Abbreviations

The following abbreviations are used in this manuscript:

AE	Adverse event
AGA	American Gastroenterological Association
BMP	Bone morphogenetic protein
CD	Crohn’s disease
CKD	Chronic kidney disease
DMT1	Divalent metal transporter 1
EHA	European Hematology Association
Fe^2+^	Ferrous iron
Fe^3+^	Ferric iron
FeSO_4_	Ferrous sulfate
GI	Gastrointestinal
Hb	Hemoglobin
HCP1	Heme carrier protein 1
HCT	Hematocrit
HF	Heart failure
IBD	Inflammatory bowel disease
ID	Iron deficiency
IDA	Iron deficiency anemia
IL-1β	Interleukin-1 beta
IL-6	Interleukin-6
IPS	Iron protein succinylate
IV	Intravenous
JAK	Janus kinase
MCH	Mean corpuscular hemoglobin
MCHC	Mean corpuscular hemoglobin concentration
MCV	Mean corpuscular volume
NHANES III	National Health and Nutrition Examination Survey III
PK	Pharmacokinetic
PPI	Proton pump inhibitor
RBC	Red blood cells
ROS	Reactive oxygen species
SI	Serum iron
STAT3	Signal transducer and activator of transcription 3
TIBC	Total iron-binding capacity
TNF-α	Tumor necrosis factor-alpha
TSAT	Transferrin saturation
UC	Ulcerative colitis
WHO	World Health Organization
